# The Incidence and Treatment of Disease

**Published:** 1918-11-09

**Authors:** 


### LONDON'S HEALTH.

### I.—The Incidence and Treatment of Disease.

IN his annual report for 1917,* Dr. Hamer deals in a masterly and comprehensive manner with the public health of the administrative county of London, and with the work of school medical inspection during the year. The report consists of two parts, the first dealing with public health, and the second with school medical inspection. In his introductory remarks Dr. Hamer refers to the interesting question of the possible relationship between the continuous heavy gunfire on the Western Front and the high rainfall in South-East England. He quotes Mr. F. J. Brodie as follows: "In 1914-17 England, S.E., stands out boldly as the wettest part of the country, with an excess of rainfall amounting to no less than 26 per cent.," but he (Dr. Hamer) wisely remarks that whether this high rainfall is due to gunfire or not, it is partly responsible for some of the comparatively low death-rates of London during the past three years.

### VITAL STATISTICS.

In common with other parts of the country, there was a sharp fall during 1917 in the civilian population and in the number of marriages and births. The civilian population is estimated by the Registrar-General at 4,026,991. The marriages registered numbered 39,400 compared with 43,855 in 1916, and the number of births was 80,550, as against 99,336 in 1916, the estimated birth-rate being 17.9, compared with 21.5 for the previous year. The death-rate showed a rise of one per thousand, being 15.7 compared with 14.7 for 1916. This rise was chiefly accounted for by the prevalence of measles and the increased number of deaths among persons over sixty-five years of age, in consequence of the cold weather during the first quarter of the year. The infant mortality was 104 per 1,000 births, while the corresponding rate for 1916 was 89. The greatest number of infant deaths occurred during the first quarter of the year, when the weather conditions were severe. In discussing the causes of infant mortality, Dr. Hamer refers to Dr. Brend's views that the most important factor in causing an excess of infant mortality in urban over rural districts is possibly a polluted state of the atmosphere, and he points out that against this view is the fact that in the City, where no wind blows which has not passed over an air-polluting area, the infant mortality is relatively low, and that the explanation of this is that the number of persons per room in tenements is lower in the City than in other central districts.

### FEW INFECTIOUS DISEASES.

The prevalence of infectious diseases was low during 1917, with the exception of diphtheria and cerebro-spinal fever. There was no case of small-pox during 1917, but in the spring of this year an outbreak, spreading from an unrecognised case which was diagnosed as pneumonia, and comprising thirty-five cases, occurred in London. One case of typhus was notified during the year. During 1917 there were 446 cases of typhoid fever, compared with 461 in 1916. In 183 cases particulars as to the possible source of infection were obtained, the chief sources being fish, shellfish, etc., and contact infection. In some eleven or twelve London cases the probable source of infection was oysters consigned directly from a polluted source. Two outbreaks of a serious illness of a dysenteric nature occurred, and, in addition, twelve deaths were registered as being due to dysentery.

* *Report of the Medical Officer of Health and School Medical Officer of the Administrative County of London.*

### EPIDEMIOLOGICAL CHARACTERISTICS OF INFLUENZA.

Dr. Hamer discusses at considerable length, and in a very persuasive and informing manner, the epidemiological relations between influenza, cerebro-spinal fever, poliomyelitis, and polioencephalitis. In relation to his thesis he lays emphasis on the importance of the nervous type of influenza, and he points out that, epidemiologically, influenza has always had the three following striking characteristics: (1) Its "posting character," involving large areas of the inhabited globe, and prevailing in each affected community for some three or four months; (2) its power of impressing the mind of observers as being a new disease; and (3) its protean manifestations. Dr. Hamer epitomises his extended argument as follows: "After fifteen or twenty years the prevalence again becomes widespread, and it is possible now, in the light of study of the intervening 'trailing epidemics' and of some knowledge of epidemiology, to recognise the cloven hoof. Thus the sweating sickness of the middle ages, the dengue of the tropics, the cerebritis, encephalitis, brain fever, etc., outbreaks of the older records, are all extraordinarily true to one or other type of influenza, and all manifest the three main characteristics above described." Dr. Hamer's views, interesting and suggestive though they be, are not likely to find ready acceptance with bacteriologists, but they will no doubt stimulate the further epidemiological study of these diseases.

### INCREASED MORTALITY FROM PULMONARY TUBERCULOSIS.

In London as elsewhere there has been a marked increase in the number of deaths from pulmonary tuberculosis. During 1917 there were 6,908 deaths from this disease, compared with 6,491 in 1916, while the number of deaths from tuberculosis other than pulmonary was 1,575, compared with 1,514 for the previous year. This increase in the deaths from tuberculosis of the lungs has been especially evident amongst the inmates of lunatic asylums. During fifty years there has been a persistent fall in the death-rate from pulmonary tuberculosis in London, the first indication of an arrest in the decline being in 1914, the first year of the war. Dr. Hamer discusses the probable causes of the rise in the death-rate from tuberculosis, and refers to the hardship and exposure of war, the lack of food, and the disturbance of the normal dietary. One predisposing cause is increased mental anxiety and worry, which exercise a powerful influence in diminishing normal resistance to disease, while the lighting restrictions have no doubt lowered the standard of house ventilation.

### DISPENSARY TREATMENT OF TUBERCULOSIS.

The scheme for the treatment of tuberculosis in London adopted by the County Council in May 1914 has been continued during 1917. The responsibility for the dispensary treatment is placed on the Borough Councils, which receive financial assistance from the County Council to the extent of 25 per cent. in respect of the cost of the treatment of uninsured persons. The London Insurance Committee makes provision for dispensary treatment and for treatment in residential institutions in respect of insured persons. The County Council has made arrangements for the use of 231 children's beds and 100 adult beds in voluntary institutions, and for such beds as are available in the institutions of the Metropolitan Asylums Board. There are, in addition, twenty-nine interim tuberculosis care committees, which work in co-operation

**London's Health**—(*continued*).

with the Council and which are chiefly concerned with the home life of the patients, the provision of extra nourishment, and the finding of suitable employment.

Venereal Diseases.

The scheme for the diagnosis and treatment of venereal diseases for London and the Home Counties came into force in January 1917. Twenty-two clinics were established in connection with London hospitals, and during the year 15,385 patients made use of the clinics, and their attendances totalled 120,659. Of this number 10,329 were London patients with venereal disease who were dealt with for the first time. The arrangements for 1918 include the provision of hostels for certain persons who required greater privacy while undergoing treatment. Consideration was also given during the year to the important question of providing treatment for infected pregnant women, but the authorities of the large lying-in hospitals who were approached on the subject were unable, in consequence of the shortage of staff and the necessity for additional accommodation, to offer the necessary facilities. The scheme of the London County Council and the participating authorities provides adequate facilities for the diagnosis and treatment of venereal diseases, and since its inception it has worked well and has given good results. Its success is assured provided that full advantage be taken of the modern methods of treatment it offers by those who are infected with venereal disease, but who too frequently shirk the question of immediate and appropriate treatment.

